# A Miniaturized FSS Using the Parallel LC Resonant with Angular Stability

**DOI:** 10.3390/s25164931

**Published:** 2025-08-09

**Authors:** Chao Sun, Guangyi Heng, Yuhang Zou, Dongmin Zhang, Chen Chen, Jiahui Fu

**Affiliations:** 1 The National Key Laboratory of Complex Aviation System Simulation, Southeast China Institute of Electronic Technology, Chengdu 610036, China; sccd2025@126.com (C.S.); zhangdongmin2021@163.com (D.Z.); chenchen4990@163.com (C.C.); 2 School of Electronics and Information Engineering, Harbin Institute of Technology, Harbin 150001, China; 24s005004@stu.hit.edu.cn (G.H.); zouyuhang2000@foxmail.com (Y.Z.)

**Keywords:** frequency-selective surface, equivalent circuits, LC resonator

## Abstract

This paper proposes a highly symmetrical miniaturized, frequency-selective surface (FSS) based on LC parallel resonance to optimize high-frequency passband characteristics, enhancing transmission efficiency under large-angle conditions. Through meandered design optimization, the device size is further reduced. Utilizing cell bending techniques and LC resonators, a single-layer FSS unit with parallel LC resonance is designed, achieving reflection and transmission peaks at approximately 1.56 GHz and 1.94 GHz, respectively. By employing co-planar and hetero-planar configurations to manipulate the effective capacitance through structural design, the reflection resonance frequency is effectively shifted beyond 0.7 GHz while preserving passband stability. The single-polarization characteristic is enhanced through cell arrangement. Experimental results validate the FSS’s transmission performance in the 1.71–2.2 GHz band under large-angle incidence (0–60°), with gain reduction not exceeding 1.2 dB. With a compact footprint (0.134λ × 0.134λ), a simple structure, and a stable angular response, the proposed FSS demonstrates strong potential for base station applications that require multi-band compatibility and spatial efficiency.

## 1. Introduction

In recent years, the ongoing advancements in wireless communication technology, particularly with the emergence of the Internet of Things (IoT) and 5G technologies, have led to increased congestion within the low-band wireless spectrum and a growing scarcity of spectrum resources. This situation necessitates the development of antennas that demonstrate improved frequency selectivity while simultaneously achieving wideband and multi-frequency capabilities.

Traditional 3G/LTE systems typically incorporate both low- and high-frequency antenna arrays. The low-frequency band is advantageous due to its excellent propagation characteristics, which facilitate extensive area coverage. In contrast, the high-frequency band offers increased capacity in densely populated areas because of its greater available bandwidth. Effective isolation between these antenna arrays is essential to minimize intra-system interference and enhance both base station capacity and coverage efficiency. This requirement necessitates the implementation of filtered antenna designs, with this research specifically focusing on achieving isolation between the low-frequency band of 0.69–0.96 GHz and the high-frequency band of 1.71–2.17 GHz.

A Frequency Selective Surface (FSS) refers to a periodic array structure consisting of metallic patch elements on a dielectric substrate or aperture elements within a metallic layer, which manipulates the reflection and transmission of electromagnetic waves for filtering applications [1]. Typically, patch-type FSS exhibit band-stop characteristics, while aperture-type FSS exhibit band-pass behavior. In practical applications, a hybrid design combining both types is often adopted to achieve the desired filtering performance [2].

For the design of FSS, the wave impedance of free space in transverse electric (TE) and transverse magnetic (TM) modes varies in opposite directions with changes in the angle of incidence of electromagnetic waves. Consequently, the equivalent size will also be affected. This divergent behavior inevitably affects the transmission stability of FSS under large incident angles in dual-polarization (TE and TM) operation. To achieve wide-angle stability, the primary methods currently employed include tortuous structures [3,4,5,6], highly symmetrical structures [7,8,9,10], multi-layer coupling structures [11,12,13,14], integrated waveguide technology [15,16], and three-dimensional structures [17,18,19]. These methods aim to realize miniaturization and introduce additional transmission poles to optimize stability under oblique incidence. However, these approaches typically involve band-stop rather than band-pass structures, and most are narrowband or multi-frequency configurations. Band-stop structures can generally achieve wide stopbands and multi-band rejection with large-angle stability. In contrast, band-pass structures can usually maintain a stability angle of only 30° without causing bandwidth distortion. For example, the configuration proposed in [20] achieves effective decoupling between dual-band antenna elements within a 20° angular range through the synergistic integration of stopband and passband characteristics. The conventional unit size of FSS is approximately 0.5λ. Current miniaturization techniques have successfully decreased this size to approximately 0.1λ or even less. Nevertheless, overly aggressive designs often result in increased complexity, which may adversely affect angular stability. Furthermore, in dual-band configurations, the wavelength at the lower frequency is longer. Consequently, at the same scaling ratio, the reduction in overall structural dimensions is more pronounced, which facilitates the integration of base station antenna structures.

This paper proposes a highly symmetrical, miniaturized FSS design based on an LC parallel unit, which adjusts the low-frequency stopband and high-frequency passband while enhancing transmission efficiency under large-angle dual polarization. Through symmetry optimization and bending design, the device size is further minimized, facilitating dual polarization. Additionally, corresponding antenna arrays were independently designed to assess key high-frequency transmission performance, thereby validating the efficacy of the FSS in optimizing the transmission characteristics of the high-frequency band in base station antennas. The empirical results align with simulation predictions, demonstrating exceptional transmission performance.

## 2. Structure Description and Operating Principle

### 2.1. Design and Analysis of the Parallel LC Resonators

Miniaturizing the FSS structure is essential to mitigate angular sensitivity and enhance oblique incidence performance. Cell bending technique and lumped element loading are two commonly used methods. They reduce the resonant frequency of the structure by bending the basic FSS unit structure to increase the resonant path length or loading inductor/capacitor elements to adjust the equivalent circuit parameters.

**Figure 1:**
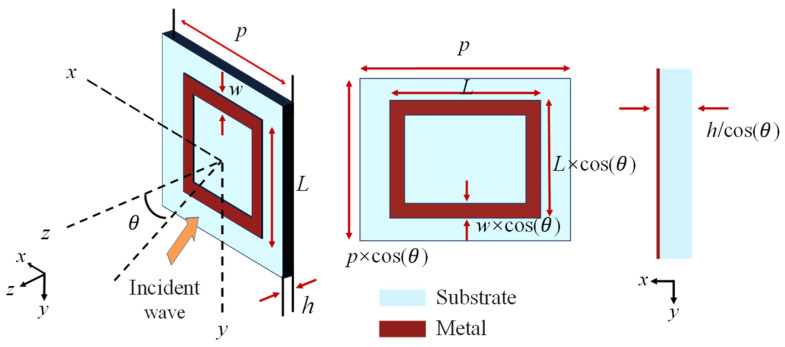
Equivalent structure of FSS under oblique incidence.

This paper primarily employs the cell bending technique. As illustrated in Figure 1, the incident electromagnetic wave in space is obliquely incident on a band-stop type FSS. The incident electromagnetic wave is parallel to the yoz plane and forms an angle of *θ* with the xoz plane. Figure 1 also depicts the FSS in the scenario where the electromagnetic waves are vertically incident (yoz plane), representing the equivalent structure of the FSS when the electromagnetic wave is obliquely incident. Due to the angle *θ* between the electromagnetic wave and the xoz plane, the length of the metal patch along the y-axis is equivalent to *L* × cos*θ*, and the thickness of the dielectric substrate increases from the original height *h* to *h*/cos*θ*. By contrast, the electromagnetic wave has no angle with the yoz plane. The equivalent length of the metal patch along the x-axis remains *L*, and the patch width *w* is the same. Consequently, to enhance the performance under oblique incidence, it is essential to minimize the structure as much as possible.

**Figure 2:**
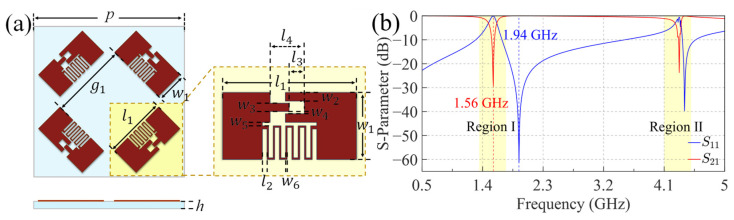
The structure of parallel LC resonators. (**a**) Top view of periodic structure; (**b**) S parameter.

Modern communication systems predominantly operate within the 1–2 GHz frequency band, requiring FSS elements with relatively large physical dimensions. Inspired by the configuration presented in [20], we used a compact single-layer FSS unit featuring parallel LC resonators, which facilitates simultaneous size reduction and the generation of a transmission pole. As illustrated in Figure 2a, the proposed structure comprises an upper metallic patch layer and a lower FR-30 dielectric substrate with a thickness *h* of 0.8 mm (*ε_r_* = 3, tan*δ* = 0.0014).

**Figure 3:**
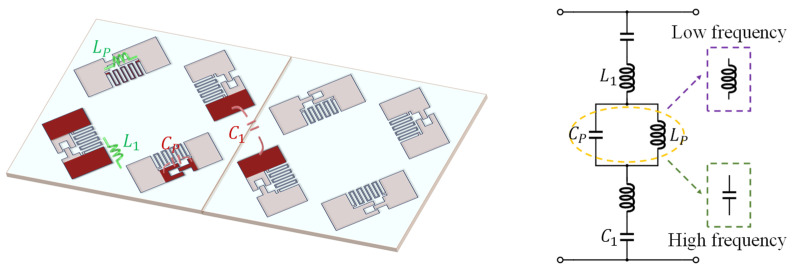
Equivalent circuit model (ECM) of FSS.

The metallic patch integrates three functional components: lateral metallic strips acting as inductor *L*_1_, a central meandered line serving as parasitic inductor *L_P_*, and an interdigital capacitor implementing parasitic capacitance *C_P_*. Full-wave electromagnetic simulations demonstrate the transmission characteristics shown in Figure 2b, revealing distinct reflection and transmission peaks at approximately 1.56 GHz and 1.94 GHz, respectively. From the equivalent circuit perspective, as shown in Figure 3, the parallel configuration of *L_P_* and *C_P_* produces an equivalent impedance *Z_P_* expressed as follows:

(1) $$Z_{P}=\frac{j\omega L_{p}\cdot \frac{1}{j\omega C_{p}}}{j\omega L_{p}+\frac{1}{j\omega C_{p}}}=\frac{j\omega L_{p}}{1-\omega^{2}L_{p}C_{p}}$$

When the operating frequency f coincides with the resonant frequency $\;f_{t}$, the parallel LC impedance *Z_P_* approaches infinity, effectively suppressing induced currents on the metallic patch surface. This phenomenon results in a broad transmission band in the S_11_ spectrum. At frequencies below or above $f_{t}$, the parallel LC circuit exhibits behavior characteristic of inductive or capacitive elements. Combined with the capacitance *C*_1_ between adjacent metallic patches, this configuration introduces two transmission zeros that generate two stopbands near the new resonance point (highlighted yellow regions in Figure 2b). The capacitive behavior of the parallel resonant branch at higher frequencies degrades the quality factor (*Q*-factor) of the series resonance, thereby compromising the stopband $f_{r2}$ rejection performance. Consequently, the analytical focus shifts to Region I within the lower-frequency stopband $f_{r1}$, which exhibited significantly stronger rejection levels. The stopband $f_{r1}\;$ was embedded within the original 1–4 GHz transmission band created by the parallel resonance in, ultimately demonstrating band-rejection filter characteristics.

**Figure 4:**
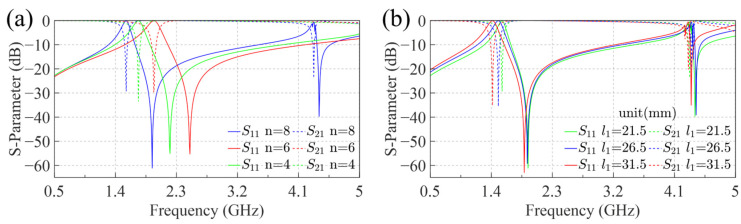
Transmission parameters on different numbers of meandered-line turns and metallic patch lengths: (**a**) the number of meander turns; (**b**) metallic patch length.

While the emergence of the stopband narrows the transmission bandwidth, it conversely provides design flexibility in positioning the transmission windows within the desired frequency range. Equivalent circuit analysis reveals that the resonant characteristics are primarily governed by the meandered line geometry and the interdigital capacitor design, with the former exhibiting greater sensitivity to structural variations in this configuration. Furthermore, the physical length of the metallic patch is a critical parameter that influences the electrical length. To systematically investigate these dependencies, parametric simulations were conducted by varying both the number of meandered-line turns and the length of the metallic patch. The resulting transmission parameter responses are shown in Figure 4.

As evidenced in Figure 4a, tuning the number of meander turns (*n*) directly modulates the parasitic inductance *L_P_*, thereby significantly altering both the two reflection resonant frequencies and the transmission resonant frequency. However, selecting a higher transmission resonant frequency partially negates the miniaturization advantages afforded by the meander-line design. Furthermore, the lower $f_{t}$ places the reflection and transmission bands in closer frequency proximity, tightening the passband constraints.

The parallel resonance configuration inherently isolates the transmission band characteristics from variations in other circuit parameters. This observation is corroborated by Figure 4b, where adjustments to the metallic patch length (*l*) induce only minor shifts in $f_{t}$. Such parametric decoupling enables independent optimization of the passband resonant frequency, bandwidth, and overall cell miniaturization. On this basis, to achieve improved passband performance and allow control over $f_{r1}$, it is necessary to increase the capacitance of *C*_1_ during the design process.

### 2.2. Analysis of Equivalent Circuits

**Figure 5:**
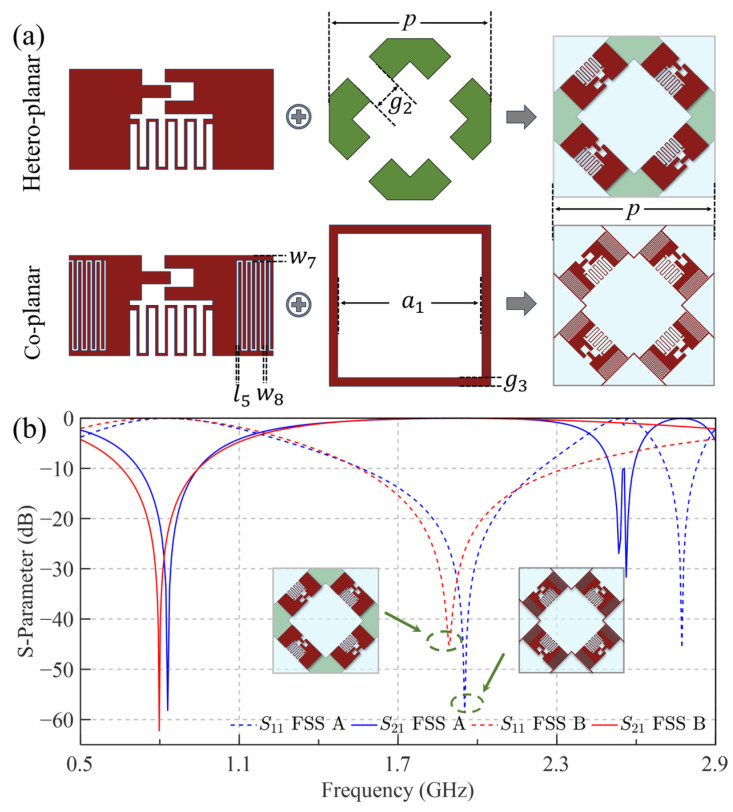
The adjustment of the reflection resonant frequency via co-planar and hetero-planar structures: (**a**) The structure of the two methods; (**b**) S-parameter.

This study achieves effective control of the capacitance *C*_1_ through co-planar and hetero-planar structural configurations, thereby suppressing undesired reflection resonance frequencies. Figure 5a illustrates these configurations, where red represents the top metal layer and green indicates the bottom metal layer, while Table 1 provides detailed geometric parameters. Considering structural stability, the co-planar configuration (FSS A) modifies the metallic patch layer by integrating interdigital capacitors and a metal loop, thereby enhancing *C*_1_ through adjacent finger coupling. In contrast, the hetero-planar configuration (FSS B) utilizes parallel-plate capacitance generated between vertically stacked square patches separated by dielectric layers. Simulation results in Figure 5b demonstrate that both configurations effectively reduce $f_{r1}$ to approximately 0.85 GHz. However, they exhibit distinct transmission characteristics: the co-planar structure maintains the original transmission frequency $f_{t}$ with a narrowed bandwidth, while the hetero-planar configuration preserves the bandwidth but experiences a small frequency shift.

**Figure 6:**
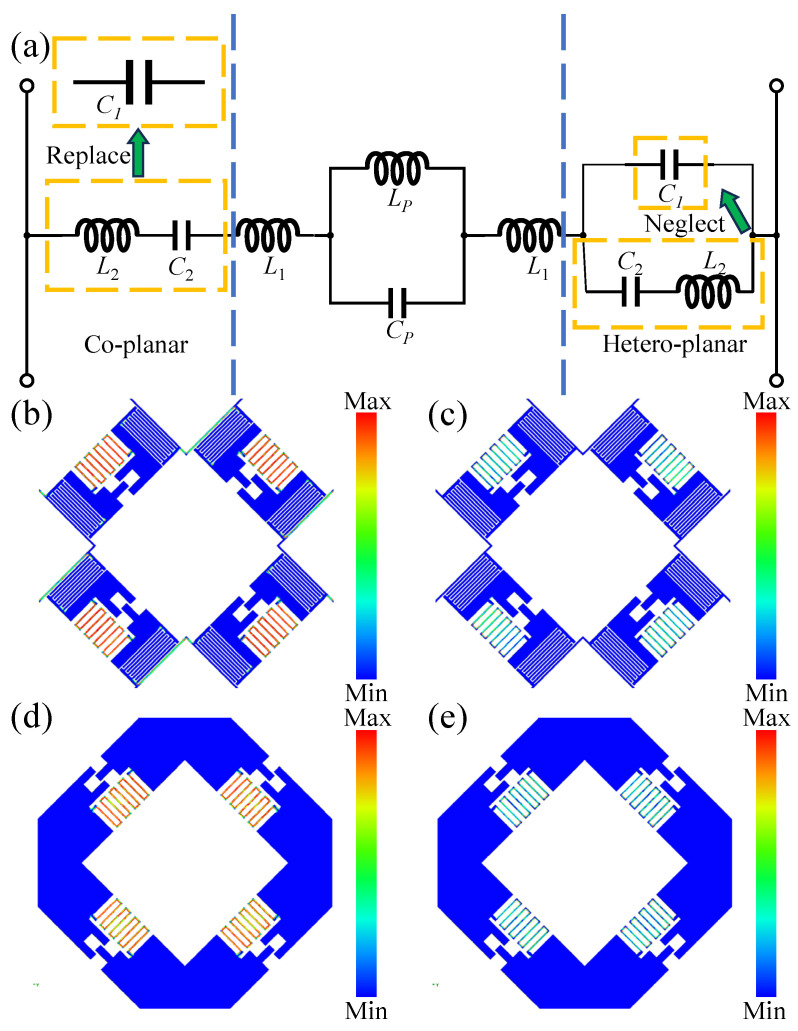
Equivalent circuit models and simulated surface current distributions for the co-planar and hetero-planar FSS structures. (**a**) The equivalent circuit; (**b**) co-planar structure at the reflection resonance frequency; (**c**) co-planar structure at the transmission resonance frequency; (**d**) hetero-planar structure at the reflection resonance frequency; (**e**) hetero-planar structure at the transmission resonance frequency.

Equivalent circuit analyses were conducted for both approaches. The co-planar design stems from the introduction of a metal loop to overcome the inherent geometric constraints of the parallel LC resonator (PLCR) unit, which limits inter-element spacing and capacitance *C*_1_. The added metal loop directly alters *C*_1_, replacing it with a new series combination of a slightly enhanced capacitance formed between the loop and PLCR and the loop’s intrinsic inductance *L*_2_, thereby lowering the resonant frequency. However, the metal loop alone cannot provide sufficient capacitance, necessitating the addition of an interdigital structure on the PLCR. This structure connects to the loop and generates a larger effective capacitance *C*_2_. The hetero-planar structure retains the original configuration by establishing an interlayer conductive path composed of its intrinsic inductance *L*_2_ and a large parallel-plate capacitance *C*_2_, connected in parallel with *C*_1_. Since *C*_1_ is relatively small, its effect is considered negligible and thus neglected in the equivalent circuit shown in Figure 6a.

Subsequent investigations focused on the current distributions at resonant frequencies. As shown in Figure 6, the interdigital structures in both configurations exhibit strong current flow at the low-frequency reflection resonance and diminished current at the high-frequency transmission resonance, corresponding to the “on” and “off” states of the equivalent circuit. Notably, in the co-planar design, the narrow metal loop connecting adjacent PLCR units shows a clear generation of current at the reflection frequency in Figure 6b. Two additional factors have emerged that warrant further consideration: (1) the interdigital structure modifies the original inductance *L*_1_, resulting in a significant reduction of the higher resonant frequency $f_{r2}$; (2) the interlayer capacitance introduced in the hetero-planar configuration perturbs the PLCR, slightly lowering the transmission frequency $f_{t}$.

**Figure 7:**
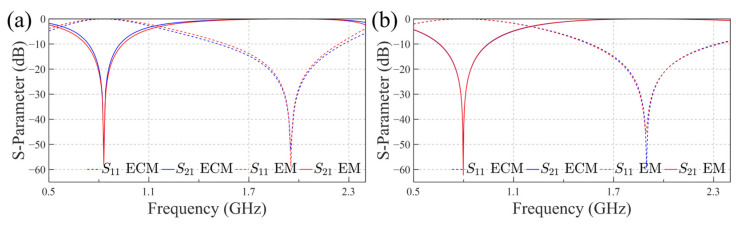
S-parameters of the co-planar and hetero-planar structures based on electromagnetic (EM) and ECM simulations: (**a**) The co-planar structure; (**b**) The hetero-planar structure.

The two proposed equivalent circuits were validated using a circuit simulation software, and the corresponding simulation results are presented in Figure 7.

For the co-planar structure, the component values are as follows: the inductance *L_P_* and capacitance *C_P_* in the central PLCR structure are 9.3 nH and 0.68 pF, respectively; the combined inductance *L*_1_ + *L*_2_ is 12.4 nH, and the additional capacitor *C*_2_ has a value of 1.69 pF. For the hetero-planar structure, *L_P_* and *C_P_* in the PLCR remain similar at 9.8 nH and 0.69 pF, respectively. The small capacitor *C*_1_ is 0.35 pF, the added capacitor *C*_2_ is 2.47 pF, and the inductors *L*_2_ and *L*_1_ are 0.89 nH and 1.6 nH, respectively.

The similarity in *L_P_* and *C_P_* between the two configurations is expected, given that the central PLCR structures are identical and only the side square metallic sections differ. The larger inductance *L*_2_ in the co-planar configuration arises from the higher inductance of the ring compared to the square metal patches in the hetero-planar case. Furthermore, the smaller value of *C*_1_ is consistent with the theoretical analysis. These results further support the validity of the proposed equivalent circuit models.

## 3. Simulation Results and Discussion

### 3.1. Structural Optimization for FSS

**Figure 8:**
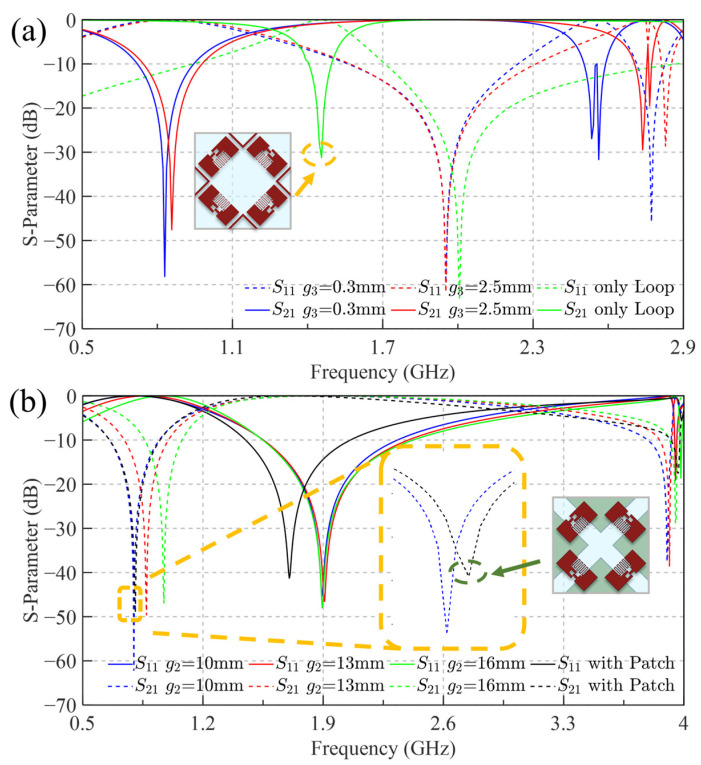
Effects of structural parameter variations on the S-parameters of the co-planar and hetero-planar designs. (**a**) Influence of metal loop width on the co-planar structure, including comparison with the only Loop structure; (**b**) Influence of inter-patch spacing on the hetero-planar structure, including comparison with the square metal patch structure.

In the co-planar design, capacitance *C*_2_ is predominantly determined by the interdigital structure and is considered fixed. Consequently, further investigation focuses on the influence of the metal loop width $g_{3}$ on the transmission parameters. Figure 8a presents transmission parameters for different values of $g_{3}$, with comparison to a configuration (green line) employing the metal loop alone, excluding interdigital capacitors. Integrating interdigital capacitors while maintaining symmetry significantly increases capacitance *C*_2_, achieving a reflection resonance near 0.85 GHz. In contrast, the configuration utilizing only the metal loop exhibits severely limited design flexibility in controlling the reflection resonance frequency, underscoring the importance of incorporating interdigital structures. Parametric studies also reveal that the transmission response $f_{r2}$ is highly sensitive to variations in loop width $g_{3}$, which in turn constrains the achievable transmission bandwidth.

The hetero-planar structure is based on the principle of a parallel-plate capacitor. Figure 8b illustrates a modified model (black line) based on FSS B, in which the bottom metal patch is extended into a full square patch, maintaining the same overlapping area as FSS B. The comparison of S-parameters between these two structures exhibits only negligible variation. This observation substantiates that the coupling capacitance *C*_2_ is predominantly governed by the overlapping area of the metal layers. Increasing the gap between patches $g_{2}$ reduces the overlap area, thereby decreasing *C*_2_ and elevating $f_{r1}$, while still maintaining it consistently below 1 GHz. Regarding the transmission resonance frequency $f_{t}$, the enlarged geometry of the square-patch structure enhances both inter-patch capacitance and cross-layer coupling effects. As the operating frequency increases, the capacitive dominance weakens, leading to deviations in the equivalent circuit behavior dominated by the large *C*_2_. These factors make the capacitive interactions between metal patches non-negligible. As a result, $f_{t}$ exhibits a noticeable frequency shift, compromising the ability to independently control the passband during the design process.

**Figure 9:**
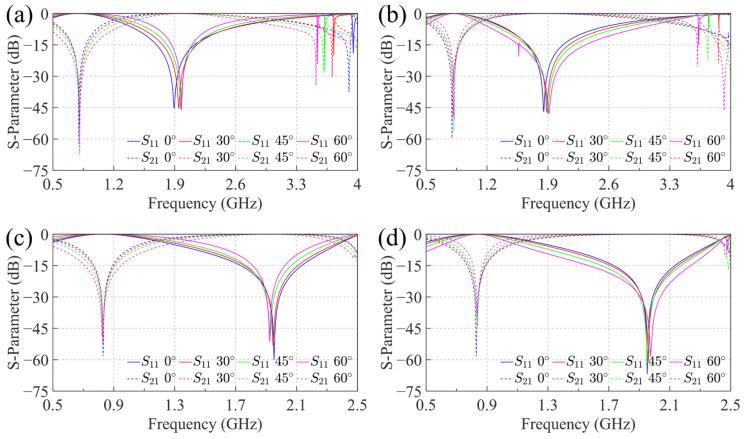
The structure of co-planar and hetero-planar FSS and the dual polarization transmission characteristics at different angles: (**a**) TE polarization of co-planar structure; (**b**) TM polarization of co-planar structure; (**c**) TE polarization of hetero-planar structure; (**d**) TM polarization of hetero-planar structure.

Both methods yield favorable results. To further evaluate their performance, we analyzed the angular stability of their dual-polarization behavior. Figure 9 presents the transmission characteristics of the two structures under TE and TM polarizations at varying incident angles.

For the hetero-planar structure, strong reflection characteristics are observed from 720 MHz to 930 MHz, with |$S_{11}$| consistently below 0.8 dB across incident angles from 0° to 60°. It also demonstrates effective transmission from 1710 MHz to 2290 MHz, with |$S_{21}$| remaining below 0.8 dB throughout the same angular range. Similarly, the co-planar structure exhibits good reflection performance from 760 MHz to 910 MHz, maintaining |$S_{11}$| below 0.8 dB for incident angles up to 60°. Its transmission band spans from 1650 MHz to 2140 MHz, with |$S_{21}$| also staying below 0.8 dB. Both structures show excellent angular stability in their resonant frequencies, particularly the co-planar design, which exhibits a maximum frequency shift of only 30 MHz.

The comparative analysis reveals that both configurations exhibit satisfactory angular stability. However, the FSS B structure offers better manufacturability and a wider 3 dB transmission bandwidth, providing greater flexibility for passband optimization. Consequently, the hetero-planar structure based on a parallel-plate capacitor was selected for prototype fabrication, with $g_{2}$ set to 10 mm.

### 3.2. Design of Antenna Array

Previous analysis of angular stability under dual polarization revealed that, as the incident angle increases, the TE-polarized passband narrows while the TM-polarized stopband narrows correspondingly. Notably, the bandwidth trends for the passband and stopband are reciprocal. Given the inherently wide passband, further improvement is challenging. Thus, efforts to enhance dual-band performance focused on expanding the stopband bandwidth under TM polarization by increasing the arrangement density of units oriented along the TM polarization direction. This approach leverages the fact that the TM passband broadens with increasing angle, minimizing any adverse effects on the overall passband performance.

**Figure 10:**
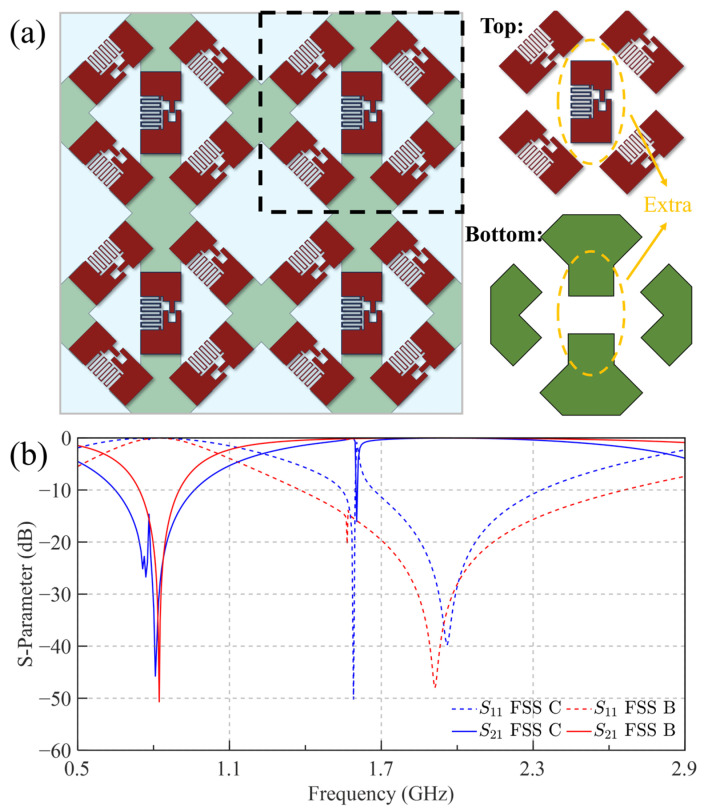
The improved unit FSS C and transmission parameters: (**a**) The structure of FSS C; (**b**) The transmission parameters of the two structures at 60°.

The resulting modified periodic structure (FSS C), incorporating an additional PLCR element diagonally for TM polarization, is illustrated in Figure 10a. Figure 10b presents the corresponding S-parameters at a 60° incidence angle before and after modification. The modified structure exhibits a significantly enhanced stopband bandwidth compared to the original design. Although S_21_ shows increased ripple, its magnitude remains consistently below –10 dB. The denser arrangement intensifies inter-element coupling, notably impacting passband performance by introducing a new transmission zero at 1.6 GHz. Communication systems typically demand high-quality transmission and reflection performance. Using a –0.8 dB criterion, the original structure exhibits a TE passband from 1.714 GHz to 2.287 GHz and a TM stopband from 718 MHz to 928 MHz. By comparison, the modified structure (FSS C) achieves an extended passband ranging from 1.637 GHz to 2.447 GHz and a significantly broadened stopband from 604 MHz to 1014 MHz. Notably, the TM passband remains sufficiently wide, surpassing the narrowest TE passband requirement, while the TM stopband bandwidth is substantially enhanced, effectively doubling from 25% to 50%.

**Figure 11:**
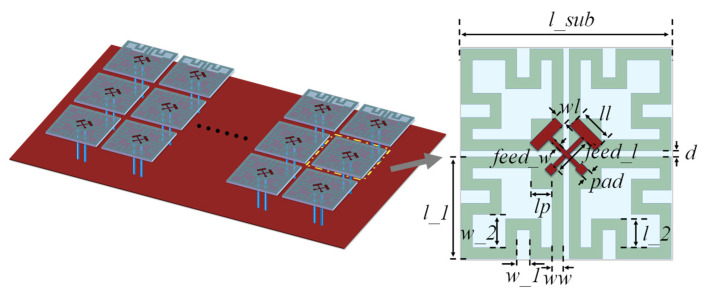
The structure diagram of the antenna array.

Finally, an antenna array consisting of 3 × 8 elements was selected to simulate a base station antenna to evaluate the actual performance of FSS C. The element spacing is 68 mm in the longitudinal direction and 55 mm in the transverse direction. Figure 11 illustrates the three-dimensional structure of the system, including the feed network integrated on the backside of the antenna array. Each antenna element radiates ±45° dual-polarized waves and is excited via a feed network composed of eight one-third Wilkinson power dividers. Detailed antenna specifications are provided in Table 2, where the antenna height *h*_2_ is 35 mm.

**Figure 12:**
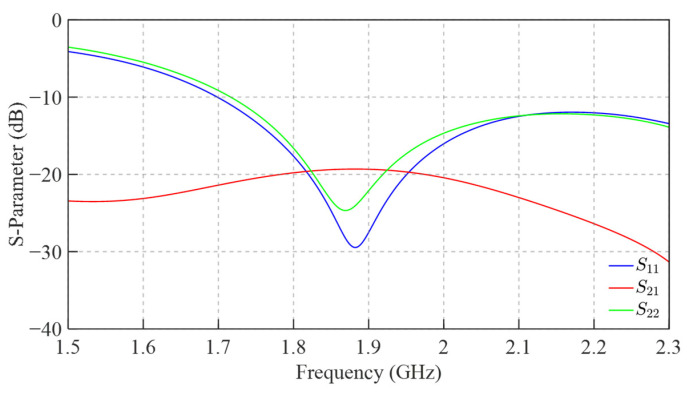
The port S-parameters of the antenna array.

**Figure 13:**
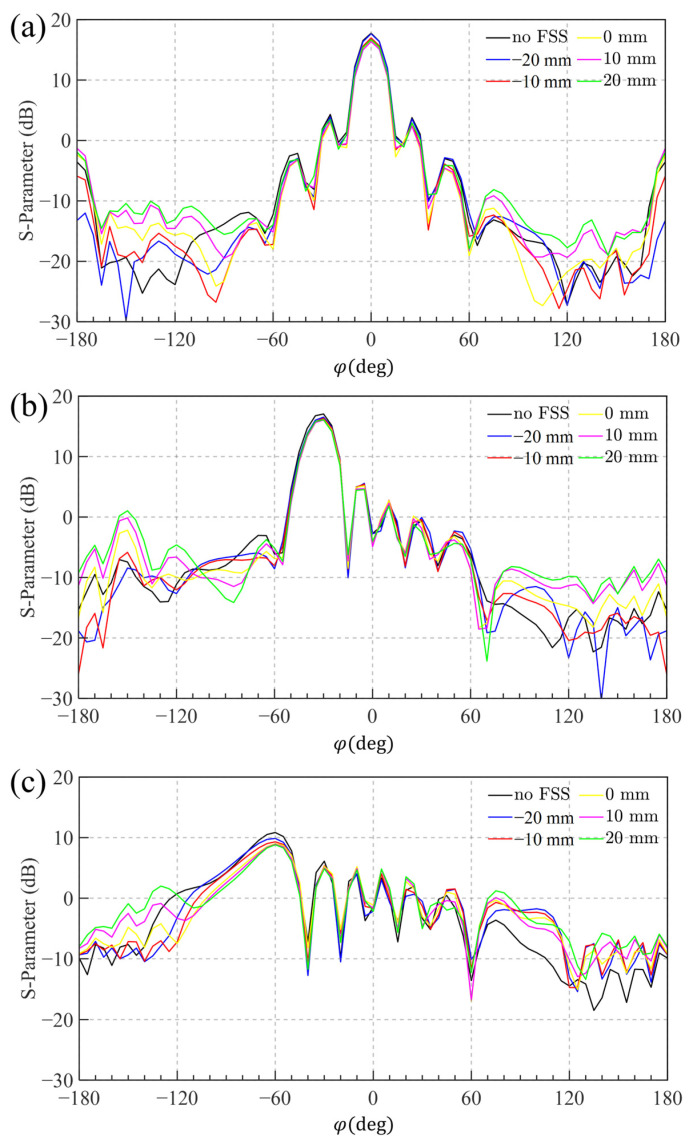
Antenna array structure and the gain pattern of the array without FSS loading and the array with FSS loading at different heights at 2.2 GHz: (**a**) Contrast of 0° beam scanning; (**b**) Contrast of 30° beam scanning; (**c**) Contrast of 60° beam scanning.

The port S-parameters of the antenna array are shown in Figure 12. The impedance bandwidth (|*S*_11_| < −10 dB) ranges from 1.71 to 2.30 GHz, with |S_21_| exhibiting a maximum value of approximately −20 dB across this band. These results align well with the bandwidth of the designed FSS. Furthermore, the entire antenna is fabricated using standard PCB processes, offering advantages of structural simplicity and ease of manufacturing. Figure 13 presents a comparison of the gain patterns between the antenna array with and without FSS loading at various heights. The initial height of the FSS was set to 50 mm. When observing the main beam, the results show that the maximum gain of the array does not decrease significantly after FSS loading, and the radiation pattern remains largely unchanged. Within the angular range of 0° to 60°, the designed FSS exhibits excellent transmission characteristics and is effectively transparent to the antenna array, with minimal impact on its radiation performance. In terms of sidelobes, a comparison before and after FSS loading indicates that the forward radiation pattern in the upper hemisphere (approximately −80° to 80°) remains essentially unchanged. However, outside this angular range, FSS loading increases the sidelobe levels in the lower hemisphere (backward radiation). Despite this, the effect does not compromise the normal operation of the base station antenna.

Furthermore, based on the comparison of six radiation patterns at different FSS heights, it is observed that lower FSS heights result in less sidelobe enhancement compared to higher FSS positions. It suggests that placing the FSS closer to the antenna is more advantageous for maintaining radiation integrity while also helping reduce the overall height and space volume of the base station system.

## 4. Prototype Fabrication and Experimental Verification

**Figure 14:**
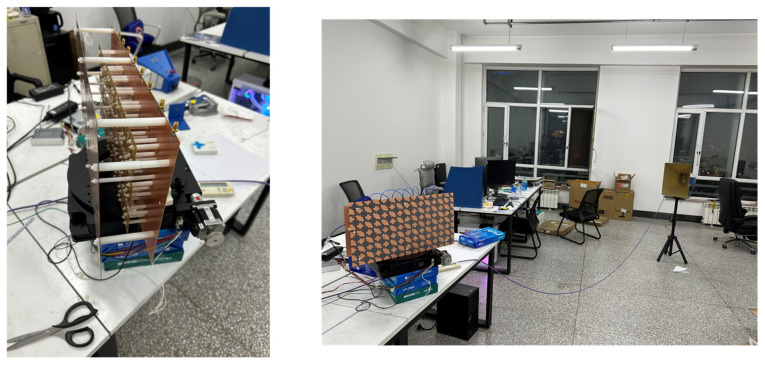
Test of array antenna loading FSS and Test environment.

To validate the FSS transmission characteristics for high-frequency base station applications, we fabricated an integrated system comprising a 3 × 8 antenna array, feed network, and FSS, as shown in Figure 14. The FSS was mounted 30 mm above the antenna array using nylon support posts at predefined positions. Eight SMA connectors embedded in the antenna baseplate were used to interface with eight-channel phase shifter modules. A motorized turntable positioned beneath the array enabled full-azimuth radiation measurements. Figure 14 shows the measurement environment used to evaluate the gain variation of the antenna array with and without the FSS under different scanning angles. The purpose of this test is to verify the effect of the FSS on the radiation performance and angular stability. The measurements were conducted in a standard indoor environment. Due to the lack of a professional microwave anechoic chamber, the test setup may be subject to some reflections and interference. Nevertheless, the test area was kept as open as possible, with large metallic objects removed from the vicinity.

A vector network analyzer (Agilent N5230C) was used as both the signal source and receiver. The transmitting antenna was the antenna array under test, and the receiving antenna was a standard horn antenna, fixed in position and aligned with the center of the array. Both antennas were placed at the same height to ensure accurate alignment. The antenna array was mounted on a motorized turntable with an angular resolution of 1°, scanning from −90° to +90° in 1° steps. The elevation angle was fixed at 0° throughout the test.

**Figure 15:**
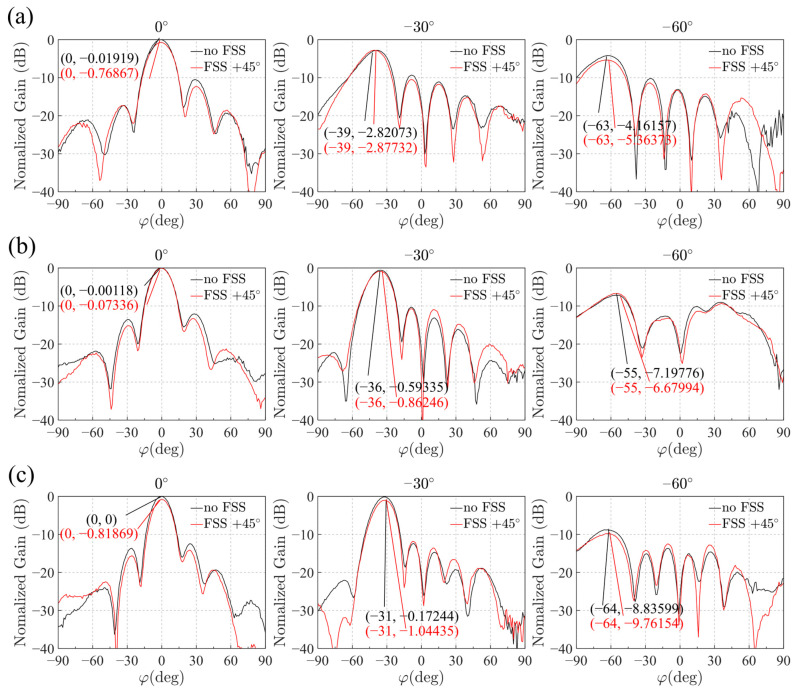
Radiation Diagram at different frequencies: (**a**) The scanning angles are 0°, −30°, and −60° at 1.71 GHz; (**b**) The scanning angles are 0°, −30°, and −60° at 1.95 GHz; (**c**) The scanning angles are 0°, −30°, and −60° at 2.2 GHz.

Before measurement, the antenna array’s phase center was visually aligned with the turntable’s center. Fine adjustments were subsequently made by comparing received power at 0° and 180° to ensure rotational symmetry and reduce mechanical misalignment effects. The measurement consisted of two sequential rounds. In the first, the antenna array was tested without the FSS, recording the power received at each rotation angle. In the second, the FSS-loaded array was tested under the same conditions. This approach ensured experimental consistency and allowed precise quantification of gain differences between the two configurations, as shown in Figure 15.

Figure 15 presents the measured radiation patterns and gain characteristics of the antenna array integrated with the optimized FSS configuration. The results demonstrate a clear improvement in performance. Due to errors in the digital phase shifter, the actual pointing angles differ slightly from the ideal settings at phase shifts of 80° (−30° pointing angle) and 140° (−60° pointing angle).

As shown in Figure 15a, at the lowest frequency of 1.71 GHz, the maximum gain difference between the unloaded and FSS-loaded array occurs at a scanning angle of −63°, with a gain reduction of approximately 1.2 dB after loading the FSS. At the center frequency of 1.95 GHz in Figure 15b, the largest gain difference appears at a scanning angle of −55°, with a gain decrease of about 0.5 dB. At the highest frequency of 2.2 GHz in Figure 15c, the maximum gain difference is observed at −64°, with a gain reduction of around 0.9 dB.

The practical test comparisons across these three frequency points show that the gain degradation after loading the FSS is greatest near the maximum scanning angles, consistent with simulation predictions. Furthermore, the gain reduction is most pronounced at the band edges, again matching simulation results. The maximum gain degradation observed is approximately 1.2 dB. Considering machining and measurement errors, the designed FSS exhibits good transmission characteristics over a wide scanning range, with gain degradation generally confined within 1 dB over the simulated angular range of 0° to 60°. High transmission and angular stability are achieved while maintaining a reasonable passband width, as detailed comparisons are presented in Table 3.

## 5. Conclusions

This work presents two dual-band FSS designs integrating LC parallel and series resonances. These structures enable flexible targeting of reflection resonance frequencies during the design stage, while maintaining favorable transmission characteristics across 1.2–2.4 GHz under dual-polarized operation, with angular stability up to 60° incidence. Through equivalent circuit analysis, we systematically decouple high-pass and stopband functionalities by balancing mutual interactions. Strategic unit cell rearrangement expands stopband bandwidth at minimal passband transmission cost. Measured results from fabricated prototypes show excellent agreement with simulations, demonstrating <1 dB insertion loss across the operational band. Featuring compact dimensions (0.134λ × 0.134λ), structural simplicity, and angular stability, this design offers significant potential for multi-band base station applications requiring spatial efficiency.

## Figures and Tables

**Table 1:** Design parameters of FSS unit elements.

Parameters	Value/mm	Parameters	Value/mm
$w_{1}$	10.65	$l_{2}$	0.85
$w_{2}$	1.35	$l_{3}$	2.45
$w_{3}$	1.3	$l_{4}$	5.5
$w_{4}$	0.35	$l_{5}$	0.3
$w_{5}$	0.55	$g_{1}$	25
$w_{6}$	0.25	$g_{2}$	10
$w_{7}$	0.5	$g_{3}$	0.3
$w_{8}$	0.4	p	50.4
$l_{1}$	21.5	$a_{1}$	13.55

**Table 2:** Design parameters of the antenna array.

Parameters	Value/mm	Parameters	Value/mm
l_1	23	ww	2.5
l_2	6.5	wl	8
lp	4.7	feed_l	9
ll	2.5	feed_w	0.83
l_sub	48	pad	2
w_1	3	d	1.4
w_2	7.5		

**Table 3:** Comparison of the proposed structure to the existing structure.

FSS Structure	Type	Unit Size	Thickness (mm)	Bandwidth Reference	Bandwidth	Operating Band Count	Angular Stability
[6]	tortuous structure	0.147λ	2	3 dB	5.6%	1	45°
[16]	Substrate Integrated Waveguide	0.48λ	1.57	1 dB	92.5%	1	50°
[17]	three-dimensional structures	0.33λ	10.4	3 dB	22.5%	1	45°
[19]	three-dimensional structures	0.18λ	12.9	3 dB	Low: 13.9% High: 10.1%	2	30°
[20]	tortuous structure	0.27λ	0.762	NA	Low: 34.1% High: 25.5%	2	20°
**This work**	**tortuous structure**	**0.134λ**	**0.8**	**1 dB**	**Low: 50.6%** **High: 25.1%**	**2**	**60°**

## Data Availability

The data are contained within the article.

## References

[1] Munk B.A. (2000). Frequency Selective Surfaces: Theory and Design.

[2] Li Z., Weng X., Yi X., Duan W., Li K., Bi M., Pan T., Lin Y. (2024). A Miniaturized Ultrawideband Dual-Bandpass Frequency-Selective Surface with High Selectivity. IEEE Trans. Antennas Propag..

[3] Ashvanth B., Partibane B., Alsath M.G.N. (2022). An Ultraminiaturized Frequency Selective Surface with Angular and Polarization Stability. Antennas Wirel. Propag. Lett..

[4] Dey S., Dey S., Koul S.K. (2022). Second-Order, Single-Band and Dual-Band Bandstop Frequency Selective Surfaces at Millimeter Wave Regime. IEEE Trans. Antennas Propag..

[5] Abidin Z.U., Cao Q., Shah G. (2022). Design of a Compact Single-Layer Frequency Selective Surface with High Oblique Stability. IEEE Trans. Electromagn. Compat..

[6] Zhou X., Sun R., Zhao P., Cao Y., Yuan B., Chen S., Wang G. (2022). A Novel Design of a Compact Frequency-Selective Surface with High Selectivity and Angular Stability. IEEE Microw. Wirel. Compon. Lett..

[7] Shukoor M.A., Dey S., Koul S.K. (2021). A Simple Polarization-Insensitive and Wide Angular Stable Circular Ring Based Undeca-Band Absorber for EMI/EMC Applications. IEEE Trans. Electromagn. Compat..

[8] Sun Z., Yan L., Zhao X., Gao R.X.-K. (2023). An Ultrawideband Frequency Selective Surface Absorber with High Polarization-Independent Angular Stability. Antennas Wirel. Propag. Lett..

[9] Dey S., Dey S. (2022). Conformal Miniaturized Angular Stable Triband Frequency Selective Surface for EMI Shielding. IEEE Trans. Electromagn. Compat..

[10] Sheng X., Wang H., Liu N., Wang K. (2024). A Conformal Miniaturized Bandpass Frequency-Selective Surface with Stable Frequency Response for Radome Applications. IEEE Trans. Antennas Propag..

[11] Bai H., Yan M., Li W., Wang J., Zheng L., Wang H., Qu S. (2021). Tunable Frequency Selective Surface with Angular Stability. Antennas Wirel. Propag. Lett..

[12] Li T., Li D., Qin P., Fan Y., Gu Y., Zuo P., Sha W.E.I., Li E. (2021). A Novel Miniaturized Strong-Coupled FSS Structure with Excellent Angular Stability. IEEE Trans. Electromagn. Compat..

[13] Chou H.-H., Ke G.-J. (2021). Narrow Bandpass Frequency Selective Surface with High Level of Angular Stability at Ka-Band. IEEE Microw. Wirel. Compon. Lett..

[14] Afzal W., Ebrahimi A., Robel M.R., Rowe W.S.T. (2023). Low-Profile Higher-Order Narrowband Bandpass Miniaturized-Element Frequency-Selective Surface. IEEE Trans. Antennas Propag..

[15] Ma T.P., Wang H.B., Cheng Y.J. (2025). A Dual-Band Dual-Polarization Frequency Se-lective Surface with Wide-Range Flexible Band Ratio and Wide Angular Stability. Trans. Microwave Theory Tech..

[16] Krushna Kanth V., Raghavan S. (2019). EM Design and Analysis of Frequency Selective Surface Based on Substrate-Integrated Waveguide Technology for Airborne Radome Application. Trans. Microwave Theory Tech..

[17] Wang P., Jiang W., Hong T., Li Y., Pedersen G.F., Shen M. (2022). A 3-D Wide Passband Frequency Selective Surface with Sharp Roll-Off Sidebands and Angular Stability. Antennas Wirel. Propag. Lett..

[18] Li D., Wu Y., Gu Z., Fan Y., An X., Chen H., Li E. (2024). Implementation of 3-D Bandstop Frequency-Selective Structures with Ul-tralarge Angular Stability Utilizing Narrow L-Shaped Strip Lines. Trans. Microwave Theory Tech..

[19] Ma Y., Zhang X., Yuan Y., Wu W., Yuan W., Huang Z., Yuan N. (2023). Dual-Polarization Multibandstop 3-D Frequency-Selective Structures Based on Cross-Coupling Method and Subcell–Cell Structure. IEEE Trans. Antennas Propag..

[20] Zhu Y., Chen Y., Yang S. (2021). Cross-Band Mutual Coupling Reduction in Dual-Band Base-Station Antennas with a Novel Grid Frequency Selective Surface. IEEE Trans. Antennas Propag..

